# The mental health of staff at violence against women organizations during the COVID-19 pandemic: Findings from a mixed-methods study of service providers in Canada’s largest city

**DOI:** 10.17269/s41997-024-00904-7

**Published:** 2024-07-29

**Authors:** Bridget Steele, Priya Shastri, Catherine Moses, Elizabeth Tremblay, Monique Arcenal, Patricia O’Campo, Robin Mason, Janice Du Mont, Maria Hujbregts, Amanda Sim, Alexa R. Yakubovich

**Affiliations:** 1 Department of Community Health and Epidemiology, Dalhousie University, Halifax, NS Canada; 2 Woman Abuse Council of Toronto, Toronto, ON Canada; 3 Toronto Region Violence Against Women Coordinating Committee, Toronto, ON Canada; 4 Dalla Lana School of Public Health, University of Toronto, Toronto, ON Canada; 5 MAP Centre for Urban Health Solutions, St. Michael’s Hospital, Toronto, ON Canada; 6 Women’s College Research Institute, Women’s College Hospital, Toronto, ON Canada; 7 Family Service Toronto, Toronto, ON Canada; 8 Department of Physical Therapy, Temerty Faculty of Medicine, University of Toronto, Toronto, ON Canada; 9 McMaster University, Hamilton, ON Canada; 10 Nova Scotia Health, Halifax, NS Canada

**Keywords:** Violence against women, COVID-19, Mental health, Staff, Mixed methods, Violence envers les femmes, COVID-19, santé mentale, personnel, méthodes mixtes

## Abstract

**Objectives:**

Staff at violence against women (VAW) organizations provide essential services for survivors of violence. The increase in VAW during the COVID-19 pandemic placed additional pressures on VAW staff. We investigated the impacts of the pandemic on the mental health of VAW staff in the Greater Toronto Area to inform recommendations for policy and practice.

**Methods:**

We conducted a community-based, mixed-methods study on the processes, experiences, and outcomes of adapting VAW programming during the pandemic using a sequential explanatory approach. Throughout 2021, we conducted a survey of direct support and leadership staff who worked on VAW services (“VAW staff”) followed by semi-structured interviews with VAW staff purposively sampled from the survey. We descriptively analyzed quantitative survey data on the mental health of 127 VAW staff. We then applied thematic analysis to qualitative data from 18 interviews with VAW staff. We used the qualitative data to support interpretation and enrich the quantitative findings regarding staff mental health.

**Results:**

In the survey, 81% of leadership and 61% of direct support staff indicated that their work was more distressing during the pandemic. Participants reported moderate symptoms of vicarious trauma and mild symptoms of anxiety and depression. We generated three themes from the qualitative data to help explain these findings: (1) challenges related to changing work environments; (2) distress over not meeting client needs; and (3) difficulties in adapting self-care strategies in response to pandemic stressors.

**Conclusion:**

VAW organizations require increased resources and flexible funding to hire and retain more staff to respond to higher and more complex caseloads during public health emergencies. With more structural supports in place, VAW organizations could create more time and space to develop their trauma-informed organizational practices: for example, establishing a culture of connection and learning among staff virtually and in-person and facilitating a range of self-care opportunities.

**Supplementary Information:**

The online version contains supplementary material available at 10.17269/s41997-024-00904-7.

## Résumé

**Objectifs:**

Le personnel des organisations de lutte contre la violence envers les femmes (VEF) fournit des services essentiels aux survivantes de la violence. L’augmentation de la VEF pendant la pandémie de COVID-19 a exercé des pressions supplémentaires sur le personnel de la VEF. Nous avons étudié les impacts de la pandémie sur la santé mentale du personnel de la VEF dans la région du Grand Toronto afin de formuler des recommandations pour les politiques et les pratiques.

**Méthodes:**

Nous avons mené une étude communautaire basée sur des méthodes mixtes sur les processus, les expériences et les résultats de l’adaptation des programmes de VEF pendant la pandémie, en utilisant une approche explicative séquentielle. Tout au long de l’année 2021, nous avons mené une enquête auprès personnel de première ligne et des cadres travaillant sur les services de VEF (personnel de VEF), suivie d’entretiens semi-structurés avec un échantillon intentionnel de ce personnel tiré de l’enquête. Nous avons analysé de manière descriptive les données quantitatives de l’enquête sur la santé mentale de 127 membres du personnel de VEF. Nous avons ensuite appliqué une analyse thématique aux données qualitatives provenant de 18 entretiens avec le personnel de VEF. Nous avons utilisé les données qualitatives pour soutenir l’interprétation et enrichir les résultats quantitatifs concernant la santé mentale du personnel.

**Résultats:**

Dans l’enquête, 81 % des cadres et 61 % du personnel de première ligne ont indiqué que leur travail était plus stressant pendant la pandémie. Les participants ont signalé des symptômes modérés de traumatisme indirect et des symptômes légers d’anxiété et de dépression. Nous avons dégagé trois thèmes à partir des données qualitatives pour aider à expliquer ces résultats : (1) défis liés aux environnements de travail changeants; (2) détresse liée à l’incapacité de répondre aux besoins des clients; et (3) difficultés à adapter les stratégies de soins personnels en réponse aux facteurs de stress de la pandémie.

**Conclusion:**

Les organisations de VEF ont besoin de ressources accrues et de financements flexibles pour recruter et retenir plus de personnel afin de répondre à des charges de travail plus élevées et plus complexes pendant les urgences de santé publique. Avec davantage de soutiens structurels en place, les organisations de VEF pourraient dégager plus de temps et d’espace pour développer leurs pratiques organisationnelles tenant compte des traumatismes. Par exemple, établir une culture de connexion et d’apprentissage entre le personnel, à la fois virtuellement et en personne, et faciliter une gamme d’opportunités de soins personnels.

## Introduction

Staff at organizations supporting violence against women (VAW) survivors provide essential and lifesaving services for women experiencing violence, including shelter and housing support, mental health and crisis support, case management, and legal advocacy. At the same time, VAW staff are at risk of experiencing emotional and psychological distress and vicarious trauma because of their work (Brend et al., 2020; Crivatu et al., 2023; Wies & Coy, 2013). This occupational stress is compounded by chronic issues of low pay, burnout, and high rates of staff turnover across a chronically underfunded sector (Beres et al., 2009; Dworkin et al., 2016; Slattery & Goodman, 2009; Wood et al., 2019). Ensuring that the mental health and well-being of staff working with VAW survivors (or VAW staff) does not suffer is paramount to effectively meeting the needs of VAW survivors.

During the COVID-19 pandemic, rates of VAW increased along with the demand for VAW support services (Piquero et al., 2021; Wathen et al., 2022; Yakubovich et al., 2023). Simultaneously, VAW organizations needed to adapt modes of service provision (e.g. switch to virtual services) to meet public health requirements, all while VAW staff were coping with new personal stressors related to the pandemic and its attendant restrictions (Wood et al., 2022; Yakubovich et al., 2023). In Canada, COVID-19 response protocols were mandated at the provincial level and changed throughout the course of the pandemic. In Ontario, the province with the most COVID-19 cases in Canada, restrictions and mandates included lockdowns or stay at home orders (including school closures), social distancing requirements, vaccine mandates, and the use of personal protective equipment (Chum et al., 2021; Shillington et al., 2021, 2022). VAW organizations had to manage and implement these rapidly evolving mandates, which, lacking the training and support of being situated within the health system, had the potential to exacerbate the impact that VAW work has on staff mental health (Garcia et al., 2021; Wood et al., 2022).

In Canada, where 44% of women over the age of 15 have experienced intimate partner violence (the most common form of VAW) in their lifetime, and where a woman or girl is murdered every 1.5 days, there has been recent increased funding for and political attention paid to preventing and responding to VAW (Cotter, 2021; Yakubovich et al., 2023). In 2022, the federal government announced CAD $601.3 million over 5 years to invest in a national action plan (NAP) to end gender-based violence, and in 2023, the NAP was released, offering provinces and territories a set of opportunities for action (Yakubovich et al., 2023). The NAP proposes that sustained funding in the sector could help with hiring and retaining qualified staff and increasing occupational health, mental health, training opportunities, and safety supports for staff. Given the increased federal and provincial funding and attention towards VAW prevention and response, identifying recommendations to improve and protect the mental health of VAW staff that address their realities is crucial to a responsive VAW service system in Canada.

Research from Australia (Carrington et al., 2021) and the United States (Wood et al., 2022) has reported on the detrimental effects of the pandemic on the VAW sector, including VAW staff mental health and well-being. In Canada, several studies have explored how VAW services were impacted by COVID-19, finding that staff at VAW organizations consistently reported increased workload and isolation (Michaelsen et al., 2022), exhaustion and burnout (Wathen et al., 2022), and difficulty separating work from home life (Mantler et al., 2023; Trudell & Whitmore, 2020). However, only two peer-reviewed studies in Canada have specifically explored staff mental health during this period. The first reported on the results of an online survey of 564 victim service providers across Canada (Roebuck et al., 2022). The authors found that 32% of participants reported a decrease in work-life balance, 42% reported that their mental health had deteriorated, and 72% reported that their level of stress had increased. The second study qualitatively examined experiences of staff working at VAW shelters and executive directors at VAW organizations during the pandemic in Ontario, Canada, and found that staff reported an increase in the emotional toll of the work, including distress, guilt, and feelings of helplessness and hopelessness with regard to meeting the needs of clients (Burd et al., 2023).

## Objectives

We aimed to understand the impacts of the pandemic on the mental health of VAW staff in the Greater Toronto Area (GTA) to inform recommendations for policy and practice. Filling the gaps in the existing evidence, we conducted the first mixed-methods study in Canada that examines the mental health of both residential and non-residential direct support and leadership VAW staff during the COVID-19 pandemic. This included the first quantitative estimates of VAW staff mental health during the COVID-19 pandemic using validated scales to assess anxiety, depressive symptoms, and vicarious trauma, which we then contextualized using a rich and novel dataset of interviews with VAW staff.

## Methods

The data used were collected as part of a community-based, mixed-methods study on the processes, experiences, and outcomes of adapting VAW programming during the COVID-19 pandemic, in collaboration with 42 VAW organizations across the GTA (the MARCO-VAW Study) (Yakubovich et al., 2023). The Unity Health Toronto Research Ethics Board (REB#20–124) and the Dalhousie University Research Ethics Board (REB# 2022–6275) approved this study.

### Quantitative data collection

Quantitative data were collected through online surveys (via REDCap) from February to April 2021. We recruited participants in partnership with VAW organizations and networks in the GTA, including through mailing lists, emails, and engagement events. All direct support and leadership staff who had been working since 11 March 2020 at an organization in the GTA with at least one VAW service were eligible to participate in the study. Survey participants had to be 18 years of age or older and able to provide informed consent. Participants received a $10 honorarium for participating in the survey.

In this study, we used participant data from the Vicarious Trauma Scale (VTS) (McCann & Pearlman, 1990; Vrklevski & Franklin, 2008), a psychometrically valid, eight-item screening tool to assess exposure to traumatic material or distressed clients, and the impact of the exposure (Aparicio et al., 2013) and we used participant data from the Patient Health Questionnaire (PHQ)–4 to assess symptoms of depression and anxiety among staff participants (Kroenke et al., 2009). We also report on whether or not staff found their work to be more upsetting or distressing during the pandemic compared to before.

### Qualitative data collection

Qualitative data were collected from April to September 2021. We purposively recruited direct support and leadership staff from the pool of survey participants who agreed to be contacted for follow-up, to broadly reflect the sample’s demographic characteristics, types of VAW services where participants worked, and the populations served. The study’s co-leads (ARY, an academic VAW researcher, and PS, a community-based VAW researcher) and three peer researchers (women with lived experience of gender-based violence who received (further) training on VAW research methods) conducted semi-structured interviews ranging from 1.5 to 2 h in length. The interview guides asked staff about their experiences delivering VAW services during the pandemic, with a specific section on their mental health. Participants provided informed consent by email prior to being interviewed and were provided with a $40 honorarium following their interviews. Interviews were conducted and recorded over Zoom (Zoom Video Communications, Inc., San Jose, CA, USA) and transcribed verbatim using Trint (Trint Ltd., Toronto, ON, Canada). BS and ARY de-identified and checked the accuracy of the transcripts to the original recordings.

### Analysis

We descriptively analyzed participant responses to the survey questions related to their mental health using Stata (StataCorp, 2017). The results of the quantitative analysis were used to inform the development of questions around vicarious trauma and mental health used in the qualitative interview guide. For the qualitative data, we used a reflexive thematic analysis methodology that recognizes and embraces the subjectivity and positionality of the research team, allows for flexible coding practices, and emphasizes iterative, in-depth engagement with the data, to identify themes related to staff mental health throughout participants’ entire transcripts (Braun & Clarke, 2019). Our analysis was informed by a critical feminist lens, accounting for systemic, societal undervaluing of work in fields that are predominately comprised of women and aiming to identify the ways in which participants’ self-reported experiences can be used to identify structural gaps in the VAW support sector and to inform policy and practice (Grimshaw & Rubery, 2007; McPhail et al., 2007).

A team of four trained researchers, three of whom participated in interviews, and two peer researchers with lived experience of violence collaborated on the qualitative data analysis. The analysis team first conducted open coding on the same two interview transcripts to obtain a fulsome picture of the dataset and then the research team collaboratively compared and consolidated codes into a framework to apply to the remaining dataset. Next, each analyst was assigned a subset of transcripts for coding. Once all transcript data were initially coded, each transcript was double coded by another analyst to identify opportunities to add codes and integrate perspectives. Then, the research team met to discuss additions and changes to the coding framework.

For this analysis on VAW staff mental health, two researchers read through the data assigned to codes on mental health. We then integrated the results of the qualitative and quantitative data (Ivankova et al., 2006). We used the codes identified in the qualitative analysis to explore contributing elements to staff vicarious trauma and anxiety and stress to unpack the quantitative findings. Embedded within this analysis was an investigation of how mental health varied across direct support and leadership staff. We then identified and developed an initial summary of the most salient data on staff mental health and generated themes based on patterns of ideas we identified across these data. Finally, we developed a thematic framework to summarize the relationships between these patterns.

As part of our integrated knowledge translation approach, VAW knowledge users (including advocates, organizational leaders, direct support staff, and women with lived experience of VAW) were engaged as active participants throughout the research process, as co-lead, team members, or advisory group members. In addition, the team regularly presented and discussed progress and preliminary findings with knowledge users across the VAW and allied sectors in the GTA through knowledge sharing events. Attendees provided input and feedback on the direction of the research and offered contextual insights to inform and nuance our data collection and analysis.

## Results

### Participants

Table 1 summarizes the sociodemographic data of the survey sample and the interview participants. Of the 127 staff respondents to the survey, 104 responded to a question on mental health. Of these participants, 71 were direct support workers and 33 held leadership roles. The average age of participants was 42 and most participants identified as a ciswoman (92%) and as heterosexual (83%). Just over half of the sample (51%) identified as an ethnic or racial minority and 42% were born outside of Canada. Over half (55%) of staff worked in residential VAW services (i.e. shelter). Half of the direct support participants and most leadership (82%) participants conducted some in-person work during the pandemic, with staff at residential organizations conducting more in-person work than staff at non-residential organizations. The supplementary material describes the diversity of programs, services, and organizations where staff participants worked.

**Table 1:** Sociodemographic characteristics of the sample

Sociodemographic characteristic	*N* (%) or *M* (IQR)	*N* (%) or *M* (IQR)
	Survey (*N* = 104)^a,b^	Interviews (*N* = 18)
Age, years	42 (31–50)	47 (40–56)
Ethno-racial identity		
White	46 (49%)	7 (36%)
Black	21 (22%)	4 (21%)
Latino/Latina	8 (9%)	3 (16%)
South or Southeast Asian	12 (12%)	2 (11%)
Middle Eastern	3 (3%)	2 (11%)
Jewish	2 (2%)	1 (1%)
Indigenous	2 (2%)	0 (0%)
Gender identity		
Ciswoman	93 (92%)	18 (95%)
Cisman	4 (4%)	1 (5%)
Transgender or gender diverse^c^	4 (4%)	0 (0%)
Sexual identity		
Heterosexual or straight	83 (83%)	15 (79%)
Gay or lesbian	4 (4%)	1 (5%)
Bisexual	6 (6%)	2 (11%)
Something else (e.g. queer, not sure)	7 (7%)	1 (5%)
Country of birth		
Canada	59 (58%)	9 (47%)
Other	43 (42%)	10 (53%)
Type of VAW work		
Direct support	71 (68%)	11 (61%)
Leadership	33 (32%)	7 (39%)

*N* number, *M* mean score, *IQR* interquartile range (i.e. the 25th and 75th percentile)

^a^104 of the 127 survey participants answered at least one question on mental health

^b^The number of participants missing on each variable was as follows: ethno-racial identity, *n* = 10; gender identity, *n* = 3; sexual identity, *n* = 4; country of birth, *n* = 2

^c^Gender diverse includes any participant who reported the following gender identities: (a) fluid, nonbinary, gender queer, or agender; (b) Indigenous or other cultural identity (e.g. two-spirit); (c) transman; or (d) transwoman. We collapsed these categories to avoid possible identification of VAW staff participants due to potentially low numbers of gender diverse staff in the city’s VAW sector

The interview sample (*n* = 18) had a similar composition to the survey sample. The average age of participants was 47 and most participants identified as a ciswoman (95%) and as heterosexual (79%). Two thirds identified as an ethnic or racial minority and 52% were born outside of Canada. Slightly less than half the staff (42%) worked in residential VAW services. As with the survey sample, there was a diversity in VAW programming delivered by VAW staff.

### Quantitative findings

Both direct support and leadership VAW staff participants reported moderate vicarious trauma symptoms on the VTS as assessed by a cut-off score of 19–42 (Table 2). The mean score was 37.5 (9.8) for direct support staff and 33.2 (9.6) for leadership. Both direct support staff and leadership faced similar levels of anxiety and depression, with participants reporting on average mild symptoms of anxiety and depression on the PHQ-4 (Table 2). Direct support staff reported a mean score of 3.6 (SD = 2.7) and leadership reported a mean score of 3.4 (SD = 2.5). Most VAW staff reported that their work was more distressing during the pandemic compared to pre-pandemic (61% of direct support staff and 81% of leadership).

**Table 2:** Staff self-reported mental health

Items on staff mental health	Survey		Interview	
	Direct support (*n* = 68)	Leadership (*n* = 33)	Direct support (*n* = 11)	Leadership (*n* = 7)
Vicarious trauma^a^: 1 (strongly disagree) to 7 (strongly agree), *M* (SD)^*^				
My job involves exposure to distressing materials and experiences	5.9 (1.7)	5.0 (1.7)	5.7 (2.4)	5.9 (1.7)
My job requires exposure to traumatized or distressed clients	6.3 (1.3)	5.2 (1.7)	6.2 (1.8)	6.1 (1.5)
I find myself distressed by listening to my clients’ stories and situations	4.2 (1.6)	3.9 (1.6)	4.5 (1.5)	4.1 (2.0)
I find it difficult to deal with the content of my work	3.2 (1.7)	3.2 (1.5)	3.2 (1.7)	2.3 (1.3)
I find myself thinking about distressing material at home	4.0 (1.8)	3.5 (1.8)	4.2 (1.7)	2.3 (1.3)
Sometimes I feel helpless to assist my clients in the way I would like	4.9 (1.8)	3.8 (2.0)	4.6 (1.9)	4.3 (2.1)
Sometimes I feel overwhelmed by the workload involved in my job	4.7 (1.8)	4.6 (1.8)	5.0 (1.7)	5.1 (1.7)
Sometimes it is hard to stay positive and optimistic given some of the things I encounter in my work	4.3 (1.7)	4.0 (1.8)	3.5 (1.6)	4.0 (2.0)
Total vicarious trauma score (range, 8 to 56)	37.5 (9.8)	33.2 (9.6)	37.0 (11.5)	34.1 (11.1)
Anxiety and depressive symptoms over the last 2 weeks^b^: 0 (not at all) to 3 (nearly every day), *M* (SD)^*^				
Been feeling nervous, anxious, or on edge	1.4 (0.9)	1.0 (0.7)	1.5 (1.0)	1.3 (1.1)
Not been able to stop or control worrying	0.9 (0.9)	0.9 (0.7)	0.8 (0.9)	0.7 (0.5)
Been feeling down, depressed, or hopeless	0.8 (0.8)	0.7 (0.8)	0.6 (0.7)	1.0 (1.2)
Had little interest or pleasure in doing things	0.6 (0.7)	0.8 (0.8)	0.5 (0.5)	0.9 (1.2)
Total anxiety and depressive symptom score (range, 0 to 12)	3.6 (2.7)	3.4 (2.5)	3.5 (2.5)	3.9 (3.3)
Have you found work more upsetting or distressing during the pandemic compared to before? *N* (%)				
No, less upsetting	3 (4%)	3 (9%)	0 (0%)	0 (0%)
No change	25 (35%)	3 (9%)	3 (27%)	1 (17%)
Yes, more upsetting	43 (61%)	26 (81%)	8 (73%)	5 (83%)

^*^M mean, *SD* standard deviation

^a^Measured using the Vicarious Trauma Scale. Cut points have not been validated but the scale developers defined 8–18 as “low”, 19–42 as “moderate”, and 43–56 as “high”. The scores in the current sample are considered on the higher end of moderate

^b^Measured using the Patient Health Questionnaire (PHQ)-4. The PHQ-4 is a brief measure of the symptom burden and a screening indicator for determining whether further inquiry into clinical disorders is needed (it is not a definitive diagnostic tool when used on its own). Total scores of 0–2 are defined as “none-minimal”, 3–5 as “mild”, 6–8 as “moderate”, and 9–12 as “severe”. The scores in the current sample are considered mild

### Qualitative findings and data integration

We found that all participants, throughout the qualitative interviews, discussed mental health, not just in response to the direct interview questions on mental health. We identified three themes across direct support and leadership staff data that described how staff mental health was impacted during the pandemic: (1) challenges related to changing work environments; (2) distress over not meeting client needs; (3) difficulties adapting self-care strategies during the pandemic.

#### Challenges related to changing work environments

The pandemic altered the work environment for both direct support and leadership VAW staff. For some staff, pandemic restrictions meant that they needed to work from home, and as a result, they lost a sense of connection and community with their colleagues. Often, this left staff to cope with challenging work situations in isolation, exacerbating experiences of vicarious trauma. This was exemplified by leadership participant P68, from a large non-residential organization, who shared that while vicarious trauma “is always an issue”, historically, a common strategy used by VAW staff to cope with it was to drop into colleagues’ offices to discuss difficult cases (e.g. subtheme A in Table 3, [hereafter referred to as “Table 3 A”], leadership P68). These in-person debriefs were vital opportunities for feedback and an emotional outlet. Staff working in person also reported a loss of connection and community throughout the pandemic. For example, P43, a direct support staff, described how, because of pandemic protocols, staff no longer went in pairs to visit clients, which made debriefs “less meaningful” due to a lack of shared experiences. P115, another direct support staff, also shared how working alone during the pandemic meant that the only chances she had for in-person interactions with colleagues were during “handovers” at shift changes.

**Table 3:** Subthemes and example data under Theme 1, Changes in workplace environment

Subtheme	Participant	Excerpt
A. Loss of collegial support	P68, leadership	We can say ‘Self-care, go for a walk,’ but like, where am I going for a walk? … And it’s the fatigue factor of constantly being online and not being able to turn it off, like during a workday, let alone what you do outside your workday … Vicarious trauma is always an issue. But, as I’ve said often, if you have a particularly difficult situation with someone, a session, you can pop into your colleague’s office and just sit with her and just talk … We can’t do that right now. You have to ‘ping’ one another, ‘Do you have time? Let’s talk on Teams,’ that sort of thing. It’s very artificial. You can’t hug one another, like you just can’t. So, there’s been a lot of tears of frustration and overwhelmedness…
	P38, leadership	So when our staff has experienced vicarious trauma and in isolation away from each other, they’re not able to debrief—like they have the informal debriefing—like they would prior. They’re working outside of, like, what we would call your window of tolerance, right?
	P140, direct support	Wow. As I said before, it took me several weeks for me to actually accept the fact that I will be using my house as my office. And I live—like I have my mom that lives with me and my husband and my two young adult kids. And my mom has dementia, so having to deal with that. When in the space where I’m trying to focus on my I work and connect with my clients, has been a huge challenge. Because I never know when my mom is going to have an outburst. OK, so that has placed added pressure on me and, you know, not being able to escape out of this, to go to the office like I used to prior to the pandemic has been really, really a huge struggle for me. Because when I experience—What I’m experiencing in my home I get support from my colleagues and in person support. It’s not the same, it’s better than than much greater and better, and I feel so much more supported when I’m in the office with them rather than they trying to support me on the phone. You know, right, so, yeah
B. Lack of boundaries between home and work life	P43, direct support	Yeah, I think so. Yeah, [pause] yeah, there were some cases where you just you really felt tired afterwards. You know, not even wanting to go out. Not even wanting to go for a walk or anything, which was difficult because at least when you were in the office, you’d leave, you had a little bit of time to decompress. By the time you got home, you’re OK. Now you were just closing the door to the office and you were walking downstairs. You were walking to the bedroom if you’re working a late shift. That was hard
	P37, direct support	For me, I don’t have any privacy at home. My personality—I always work. I’ve been working for more than 20, 25 years, but I always work at the office and when I come home, it just, I put everything behind the door and I come to my unit. Now, there is no privacy for me. Mentally, I mean, all the time I’m seeing my dining table and all the files, all the equipments, and. I’m getting used to that, but kind of disturbing
	P92, direct support	Like I said, at the first, it’s very challenging to separate my work, from my home. Like to not think about a case, like a really bad case because all the cases are different. But I have cases that that like, it sticks in your mind, like all day. And go to bed and try not to think—because you think, oh my God, [where] going to do tonight. What is going to happen. What happen if I call tomorrow, like so I have to learn
C. Fears over contracting COVID-19	P15, direct support	I think just like the stress of COVID bringing it to work and. Yeah, I think that’s about it – or catching it, carrying it and bringing it home is another stressor. Yeah
	P103, direct support	So [pause] OK, so in the beginning, in the beginning. It was the most difficult, I guess, because I had people in my life who were in a panic constantly and [pause] for me even to go to work was making them even more stressed out so that stressed me out and I felt like I’m already dealing with the stress at work and I have to worry about with my family being exposed just in case if I get it and I bring it home for someone who has high risk. So that kind of emotional stress, that was really challenging for me and I had to be—it’s almost like I was a counselor at work and I was the counselor at home trying to tell them we’re going to be OK. We’re taking every precaution, nobody at the shelter got it. We’re not going to get it if we’re smart, you know, like all this talks about it. But sometimes it works, sometimes it doesn’t, you know, like you have people [pause] live in fear and then make you feel like you’re causing them that as well just because you’re going to work. However, I have to go to work. Not that it’s an option, right. Like I have to go to work because this is my job. I’m not going to lose my job because of Covid and I have a strong work ethics. I’m not going to go on sick leave because of Covid like, I feel like this is—it’s like you give up and I’m not the kind of person who will give up easily. So maybe that’s just part of my personality

Staff participants reported that they did not find the virtual environment was able to replicate what leadership participant P38 from a large non-residential organization described as “informal debriefing” (Table 3 A). For example, direct support staff P23, P43, and P68 who typically relied on support from colleagues and team members to process cases to mitigate vicarious trauma felt that this was a major missing piece resulting from the switch to virtual work. The loss of social interaction, collegial support, and community was also felt by staff who relied on in-person interactions with colleagues to help process difficult aspects of home life. Direct support staff P140 described a challenging home environment with complex care responsibilities; prior to the pandemic, she valued the in-person support and perspective she received from her colleagues in processing her home circumstances, but this stress was compounded when working from home (Table 3 A).

Many staff, like P140, indicated that an additional challenge to working from home was keeping work and home life separate. Prior to the pandemic, having separate spaces for work and home meant they were able to set healthy boundaries for when to think about work and when to disengage, as a way to manage vicarious trauma as well as symptoms of anxiety and depression. For example, direct support staff P43 shared that the lack of physical separation between home and work made it hard to decompress and detach from challenging work situations, making work all-consuming (Table 3 B). Another participant, direct support staff P37, reported feeling “disturbed” by having to see work-related files and equipment on her table. Working from home also created stressors related to client confidentiality (Table 3 B). By working in more personal spaces, oftentimes with children or family members also at home, staff experienced worry in trying to ensure that they could maintain the privacy of the clients they worked with. The consequence of not being able to have a physical separation between work and home was detrimental to staff well-being. For instance, direct support staff P92 explained that since working from home, client cases were stuck in her mind all day and night, impacting her sleep and ability to relax after work (Table 3 B).

Working from home was not the only change occurring for staff at VAW organizations. For participants whose roles required in-person work (e.g. shelter staff), the stress of managing infection prevention and control procedures, combined with fears about contracting the virus and staffing shortages, brought about anxiety. Direct support participants from residential organizations, e.g. P15 and P103, felt anxious about the possibility of being exposed to the virus in a work setting and bringing it home to their families (Table 3 C). P103 described emotional stress from worrying about infecting people close to her who had underlying health conditions while also knowing that if she did not attend work, she could lose her job.

#### Distress over not meeting client needs

VAW staff reported hopelessness and distress over feeling that they could not meet the needs of their clients. Pandemic restrictions limited their ability to support their clients in addressing their safety. Early in the pandemic, many wraparound support services for survivors (e.g. housing and legal support) reduced service capacity or temporarily shut down, leaving staff uncertain of where to refer clients and worried that clients may not receive certain timely help. For example, P5 (leadership) and P140 (direct support) felt increased stress in their jobs knowing that if they referred a client to a certain support, they might not be able to reach anyone by phone or email or receive adequate help when it was needed (Table 4 A).

**Table 4:** Subthemes and example data under Theme 2, Distress over not meeting client needs

Subtheme	Participant	Excerpt
A. Reduced availability of wraparound supports	P5, leadership	P5 [01:03:05] Well, I think it’s gotten lower. There was a point when it was really high, because, again, I was worried about how I’m going to find all these services. Where am I going to refer these women to? How do we know who’s open and not open? And because, again, we were having so many calls, for the staff to take the time to call the agencies themselves, right, because we don’t want to refer [if it’s not] helpful. Right. So for the staff to be taking the time to call those women, that’s that many more dropped calls than receiving on the line. People can’t get through. So that was actually more stressful than it is now, because even though we still, you know, even though we still have dropped calls, so even though we’d be you know, grateful that the ministry had given us funds, it’s not enough to manage the true load. Right. So we’re still pretty overwhelmed, if that makes sense. But like I said, it’s so much different, just kind of being like, you know, we’re running at, you know, basically 100 percent capacity
	P140, direct support	P140 And Outside of that personal piece. Where work is concerned because I’m so passionate about my job. It took me several weeks to actually accept. What was happening, I was becoming frustrated, I was becoming angry because I wasn’t able to support my clients who need the support desperately because so many doors were closing, you know, didn’t know like who to refer my clients to for the services that we are not offering at [organization]. You know, you’d be calling and you’d be on the on the phone holding for more than an hour. You know, the client doesn’t have time. The client is taking a chance to make a phone call and then they have to wait for an hour or more just to get somebody live on the other end of the phone. So I had to actually ground myself and say, you know what, this is real. There’s nothing you can do. This is not your fault. You just got to go with the flow and try to work with what you have until better can be done. But I am a strong advocate and I like to get things moving, and because with the pandemic, things weren’t moving the way they normally do. So I became very frustrated
B. Increased workload	P136, leadership	Yeah, we increased supervision time, but that’s – you know, who is to reduce burnout from our supervisors either, right? Like, you know, I’m not from the psych profession, so I don’t know how they reduce burnout. But hashing out on your issues, does that make it better or does it make it worse, right? Like, I’m a very result-oriented person, if the issues are not solved, then your emotions remain there, right? The fact that you can talk to someone, does it make it better, right? It only makes it better when you have more colleagues to share your work. It only makes it better when the clients are served, right? So, yeah, I don’t know how my staff feels from that. And I think they’re probably still very worked up—oh, actually overworked, yes
	P38, leadership	But, yeah, staff morale was like super, super, super low. Now, what we’re seeing is just like the impacts of trauma on our staff. And yes, this problem solving, reacting, not as being kind of on their A game like they would have before, and I think that’s a function of the trauma and the workload and things like that
	P115, direct support	I think it’s pretty obvious a lot of our staff are burnt out. It’s really hard because before we had a really great – it’s with everyone—like work life balance. And yeah, a lot of our staff are burnt out, I just think our tolerances, I don’t – I’m not saying we have poor care, I’m not going to say that, because we don’t—we’re a great, great team. Because it goes without saying vicarious trauma is taking a toll on my staff, we have one staff member in a mental health leave from all the vicarious trauma. So I don’t think right now—I think any health care professional, I want to just be very generic. I don’t think we’re giving the the top notch care that we should be providing just because the burn out It’s kind of always like, OK, well, you need to stay an extra four hours and you can’t leave, you can’t go. And it’s fine. But I think after a year of doing that, almost, I want to say once or twice I’m there till from seven a.m. until midnight, like it’s [laughs] it takes a toll. Right
C. Staffing challenges	P23, direct support	Yeah. So we have a team. It’s called the […]. So I would say we are understaffed [laughs]. But the morale is that we’re all going through very challenging times. You know, we all needed kind of to bring ourselves to speed with the technology and working with women in these situations—it really is difficult. So there’s definitely been a lot of stress and, you know, we are worried about the women’s ability to leave those situations safely. So there’s just more stress and, you know, like, scrambling for resources, like where can we send them? Like, we really try to work collaboratively. It’s, I definitely can say that the morale, we are struggling. We’re very, we’re struggling now

Increased workload and staffing challenges during the pandemic also contributed to staff feeling that they were not meeting client needs. Increased caseloads and complexity, in addition to staffing shortages, meant that some clients were not able to receive timely access to services. Staff were left overwhelmed with guilt and concern for their clients, often extending themselves to meet client need at the expense of their own mental health. Many staff reported feelings of stress and burnout due to increases in caseloads combined with understaffing. This stress was compounded for staff working at organizations that service specific communities (i.e. based on language, culture, religion, or nationality). P136, a leadership participant from a community-specific organization, described how waitlists went from 2 to 3 weeks to 6 months due to the volume of cases, and because they were the only resource in the community, they could not refer clients to any other organization, leaving client needs unmet (Table 4 B).

The relationship between increased caseload and complexity with staffing issues proved to be a vicious cycle. Aptly depicted by P115, unmanageable workloads left staff feeling exhausted and hopeless, and in some cases caused them to quit. However, these staffing shortages and turnover left an additional burden on the remaining staff to work more to try and meet client demand (Table 4 B). Ultimately, as P115 described, this cycle “takes a toll” on staff mental health and leadership participant P38 described staff morale as “super, super, super low” as a result of a deep sense of guilt and despair due to workload and burnout (Table 4 B).

#### Difficulties adapting self-care strategies during the pandemic

Having the space and capacity for VAW staff to develop and use self-care strategies is paramount for maintaining positive mental health and managing vicarious trauma. However, many VAW staff shared that because of the pandemic, they could no longer rely on the self-care strategies they typically used. P37 explained that during the pandemic, for the first time in her life, she experienced anxiety. She shared that prior to the pandemic she was effective in implementing self-care strategies (e.g. swimming) but since the pandemic she had struggled to find something equally helpful to de-stress (e.g. when pools were closed) (Table 5 A). Staff were limited in their ability to practice self-care, partly because restrictions and lockdowns prevented people from doing things that would normally provide them with breaks from work, including exercise (P37) or travelling (P115). P103 described how during the height of the pandemic, the thought of taking a vacation was unappealing, given that there was nowhere to go due to travel restrictions and very few activities open (Table 5 A).

**Table 5:** Subthemes and example data under Theme 3, Barriers to implementing self-care strategies

	Participant	Excerpt
A. Challenges adapting self-care strategies to pandemic times	P37, direct support	I got anxiety. When I talk to my family doctor last week, she assessed me and I have anxiety. I never had anxiety before. So it’s related to COVID, I guess. It’s related to this kind of work, I guess. I don’t know. But I myself, I got anxiety too. […] I used to have self-care [pause] walking every day, sometimes going swimming. And I was very good with my self care. But now I guess I don’t do the self-care. I have a dog. And if I go out, it is just because of my dog, walking her three times a day. Otherwise I don’t do the self care. It’s my bad, it’s not related to the agency or other work. No, I don’t know what’s happened to me, but yeah, there are some changes with me
	P103, direct support	Like you have your vacation so you have your leisure time so you could keep going. We didn’t have that. We just kind of—you have to keep going without that break. And at one time we were told you take a vacation or you’re going to lose it. I understand all these financial thing they have to deal with at the agency, so, yeah, you take your vacation, you sit at home and look at walls. I mean, you could watch so much TV before you go crazy and not everybody have a backyard. I mean, I was, I’m lucky I have a backyard I could sit. But you want to see people. You couldn’t, we couldn’t see people
	P95, direct support	But with certain colleagues, I have noticed that it has been a lot more challenging for them. And to be honest, again, very similar to with clients, I feel that it oftentimes, it is the colleagues who perhaps are a little bit more socially isolated because they themselves are newcomers in Canada or they’ve perhaps even just relocated from a different city and perhaps don’t have as many social ties or family that they’re actually able to spend time with. So, for example, they live alone or don’t have a partner that they live with or children or whatever the case is
B. Staff facing similar challenges to clients	P68, leadership	So we’ve tried to support people in different ways. But the ongoing, sitting in front of a screen every day in and out or on a phone, in and out every day is grinding. And so we’re really trying to work individually with people around self care, flexing time, anything that we can grab onto. It was the subject of discussion, actually, at our senior team meeting this morning again. It’s tough, you know, and, it’s tough. I don’t know what else to say. Like we’re working with clients who are experiencing social isolation and our staff are experiencing social isolation as well. So there’s. Yeah
	P137, leadership	I think staff are having a hard time, I think, across the board. Like they are tired, they’re experiencing, you know, clearly many of the same things that clients are. And I think it’s really, really hard for staff when they don’t have an answer, like, I know we can’t fix everything. I remind them quite regularly, we can’t fix everything. Yet they – it’s almost like I have to keep pulling them out of the valley of despair with the clients because it’s kind of a very slippery slope into the hopelessness, helplessness

Other staff relied on social interaction as a form of self-care, but due to working from home and pandemic restrictions, many staff experienced social isolation. P95, a direct support staff, explains how this was particularly true for people living alone, reporting that for staff who were newcomers to Canada, this loneliness was particularly challenging due to separation from family and other established support networks (Table 5 A).

Across the dataset, participants discussed how in many cases direct support staff were facing similar challenges to clients during the pandemic. P137 (leadership) described how VAW staff themselves, many of whom were on part-time or casual contracts without access to benefits, often faced increased care responsibilities, economic impacts, loneliness and isolation, and increased stress, which compounded a sense of “hopelessness and helplessness”. P137 witnessed her staff enter a “valley of despair with the clients” due to a lack of control and ability to fix the challenges facing both their clients and themselves. Leadership participant P68 similarly discussed the challenges of trying to support VAW staff with their self-care during the pandemic, sharing that there was only so much that could be done when the reality of the situation was that staff were working in isolation, in front of screens every day, and in addition to experiencing social isolation in their personal lives (Table 5 B).

## Discussion

Our mixed-methods study assessed the mental health of VAW staff in Canada’s largest city during the COVID-19 pandemic. To start, we offer the first quantitative analysis of Canadian VAW staff mental health and vicarious trauma using validated and standardized measures and previously established cut offs. First, we found that staff showed moderate vicarious trauma symptoms. The mean total scores were slightly higher than what was found in a pre-pandemic study on vicarious trauma among a sample of victim advocates working in the USA (Benuto et al., 2018) and significantly higher than a pre-pandemic study on vicarious trauma among US social workers (Aparicio et al., 2013). Second, we found that both direct support and leadership staff had mild symptoms of anxiety and depression. The total mean score from our sample was larger than the total mean scores found in general population studies before the pandemic (Kroenke et al., 2009; Löwe et al., 2010) but comparable with the mean estimate using a Canadian general population sample during pandemic times (Emodi-Perlman et al., 2021). We also found that most staff reported that their work was more distressing during the pandemic compared to prior to the pandemic.

We found that both direct support and leadership staff reported similar levels of vicarious trauma, and anxiety and depression and that more leadership staff, when compared to direct support staff, found their work more distressing during the pandemic. This analysis provides a novel contribution to research on staff in the VAW sector. Based on previous studies using samples of health care staff, we could have expected an additional burden placed on direct support staff (and not leadership), due to their direct relationships with survivors, and other job-related factors such as increased workload and lower pay (Oğlak & Obut, 2020; Trumello et al., 2020). Our qualitative data help explain this finding: many leadership staff described the feelings of responsibility, guilt, and hopelessness they experienced in being unable to offer effective solutions to the challenges faced by direct support staff in supporting survivors or managing the trauma they were experiencing from service adaptations due to the pandemic. In many ways, leadership staff felt the weight of both their organization’s clients’ increased needs and those of their staff as a result of the pandemic.

Using the qualitative data to further unpack the quantitative results, we found that staff repeatedly and consistently discussed the impact of their work during the pandemic on their mental health, providing a deeper understanding of factors contributing to mental health challenges. We identified the following three themes to describe how staff mental health was impacted during the pandemic: (1) challenges related to changing work environments, including loss of collegial support, lack of boundaries between home and work life, and fears over contracting COVID-19; (2) distress over not meeting client needs due to reduced availability of services, increased workload, and staffing shortages; and (3) barriers to implementing self-care strategies, including challenges adapting self-care strategies during the pandemic and staff facing similar personal challenges as clients.

Many of the qualitative themes we identified in our analysis, including increased workloads, reduced support between colleagues, challenges separating work from home life, and limitations to self-care strategies, were consistent with findings from similar studies on the well-being of staff working at VAW organizations (Burd et al., 2023; Nnawulezi & Hacskaylo, 2022; Roebuck et al., 2022). These parallel findings help to strengthen the evidence base on mental health among VAW staff. A unique strength to our study, however, was the diversity of our sample. In having a diverse sample, we identified novel factors influencing VAW staff mental health, including challenges faced by staff working at culturally specific VAW organizations, loneliness felt by staff who had no family living in Canada, and challenges staff faced when working from home in multigenerational households where they had care responsibilities.

While we were committed to capturing experiences from staff with a diversity of personal and social identities, most of our sample identified as heterosexual ciswomen and may not capture the unique experiences of gender and sexual minorities working at VAW organizations during the pandemic. Our study also only focuses on VAW staff from Toronto, Canada’s largest and most diverse city, with expansive health and social services. While we provide an in-depth snapshot of Toronto-based perspectives, it is necessary to replicate this research in other Canadian regions (including rural and remote areas) and in Indigenous communities.

The combination of public health restrictions and increased VAW during the pandemic created new and exacerbated existing strains on a support sector that was already at capacity and contending with what Mantler et al. (2023) aptly described as multiple pandemics (including systemic racism, poverty, and opioid-related overdoses). For decades, scholars and activists have written on how women’s organizations, including VAW organizations, have been chronically underfunded due to societal misogyny, which devalues women’s work, health, and safety (Beres et al., 2009; Knight & Rodgers, 2012; Quinlan & Singh, 2020). The creation and continued existence of VAW organizations—in the face of enormously inadequate resources—is a testament to the resilience of feminist and VAW advocacy movements. The staffing shortages, burnout, exhaustion, and vicarious trauma described in our study have plagued the sector long before the COVID-19 pandemic began (Beres et al., 2009). The new challenges posed during the COVID-19 pandemic, however, made it even more difficult for VAW staff to meet the diverse needs of clients and, ultimately, their own health and well-being needs.

VAW organizations require a substantial increase in resources and flexible funding to hire and retain additional staff to respond to increased caseloads and complexity of cases during public health emergencies, to increase the wages of staff to reduce staff turnover, to increase programmatic capacity, and to establish stronger pandemic preparedness and infection prevention and control strategies so that VAW staff can establish more robust work-life boundaries, and respond to PHE conditions with adequate training and equipment. With more structural support in place, VAW organizations could create more time and space to work with staff to develop their trauma-informed organizational practices that establish a culture of connection and learning among staff virtually and in-person and facilitate a range of self-care opportunities to prevent further trauma during emergencies (Nnawulezi & Hacskaylo, 2022).

## Conclusion

Making meaningful how the VAW sector is resourced necessitates a shift in how direct support work for VAW survivors is valued and prioritized by government and policymakers. The federal, provincial, and territorial commitments to a National Action Plan to end gender-based violence is a promising first step, but it is necessary that this funding is used to address the structural deficiencies across the VAW sector to ensure organizations and staff are prepared for success in responding to survivors’ needs while also maintaining their own health, well-being, and safety.

## Contributions to knowledge

What does this study add to existing knowledge?

- We conducted the first mixed-methods study in Canada that examines the mental health of both residential and non-residential direct support and leadership staff working at violence against women (VAW) organizations during the COVID-19 pandemic.
- We offer the first quantitative estimates of staff mental health during the COVID-19 pandemic using validated scales to assess anxiety, depressive symptoms, and vicarious trauma, which we then contextualized using a rich and novel dataset of interviews with VAW staff.

What are the key implications for public health interventions, practice, or policy?

- Direct support work for VAW survivors must be valued and prioritized by government and policy makers due to the increasing number of and severity in cases of violence against women, the critical lifesaving role staff play in the lives of survivors, and the impact of the work on staff mental health.
- This study highlights the need for VAW organizations to receive significantly increased financial and staffing resources so staff can effectively and safely respond to higher and more complex caseloads during public health emergencies.

## Supplementary Information

Below is the link to the electronic supplementary material.

Supplementary file1 (DOCX 18 KB)

## Data Availability

NA.

## Code availability

Available on request from the authors.
