# Supplementary material for: The mental health of staff at violence against women organizations during the COVID-19 pandemic: Findings from a mixed-methods study of service providers in Canada’s largest city
Source: Can J Public Health. 2024 Jul 29;115(5):756–69. doi: 10.17269/s41997-024-00904-7 (PMC11535010; doi:10.17269/s41997-024-00904-7)
Supplement: Supplementary file 1 — Supplementary file1 (DOCX 18 KB) [file 41997_2024_904_MOESM1_ESM.docx]

Description of the type of VAW work

| **Type of VAW work** | **Survey**  **N** | | **Interviews** |  |
| --- | --- | --- | --- | --- |
|  | **(N=104)** | | **(N=18)** |  |
|  | **Leadership (N=33)** | **Direct support (N=71)** | **Leadership (N=7)** | **Direct support^a^ (N=11)** |
| **VAW organization** |  |  |  |  |
| Generalist | 28 (85%) | 60 (80%) | 4 (43%) | 8 (73%) |
| Community-specific^b^ | 5 (15%) | 11 (20%) | 3 (57%) | 3 (27%) |
| **VAW service** |  |  |  |  |
| Residential | 20 (61%) | 37 (52%) | 2 (29%) | 6 (55%) |
| Non-residential | 13 (39%) | 34 (48%) | 5 (71%) | 5 (45%) |
| **VAW programming^c^** |  |  |  |  |
| Mental health, counselling, crisis support, case management | - | 30 (42%) | - | 3 (27%) |
| Shelter | - | 19 (27%) | - | 2 (18%) |
| Transitional housing support | - | 14 (20%) | - | 4 (36%) |
| Children’s Aid Society | - | 1 (1%) | - | 0 (0%) |
| Partner assault response | - | 2 (3%) | - | 1 (9%) |
| Other: |  |  |  |  |
| Healthcare | - | 2 (3%) | - | 1 (9%) |
| Harm reduction | - | 2 (3%) | - | 0 (0%) |
| Legal advocacy and support | - | 1 (1%) | - | 1 (9%) |

^a^VAW service and VAW programming do not add up to 100% as one participant worked on both transitional housing support and counselling.

^b^Community-specific organizations included language-based and culturally specific organizations.

^c^Only direct support staff participants were asked to indicate their programmatic specialization as in most cases leadership were responsible for directing, managing, or supervising an entire VAW service or organization. Specializations do not add up to 100% as one participant worked on both transitional housing support and counselling.
